# Peritoneal biopsy frozen section: cachexia manifesting as signet ring-like cells

**DOI:** 10.1515/pp-2020-0143

**Published:** 2020-11-13

**Authors:** Lakhdar Khellaf, Philippe Rouanet

**Affiliations:** Pathology Department, Institut du Cancer de Montpellier, Montpellier Cedex, France; Surgical Oncology Department, Institut du Cancer de Montpellier, Montpellier, France

**Keywords:** cachexia, fat atrophy, peritoneal metastasis, signet ring-like cells

## Abstract

Peritoneal metastases from signet ring cell adenocarcinoma may be overlooked at laparoscopy, resulting in problematic false-negative diagnoses. Conversely, false-positive diagnoses are rarely reported. For the surgeon, cachexia may rise suspicion for peritoneal metastases by exhibiting a worrisome micronodular appearance of the peritoneum, and atrophic adipocytes looks like signet ring cells at the microscopical level. Being aware of this underdiagnosed condition may help avoiding unfortunate false-positive diagnoses of peritoneal metastases during intraoperative consultation.

A 73-year-old woman underwent surgery for a gastric signet ring cell adenocarcinoma. Laparoscopic examination reported suspect micronodules of the lesser omentum (Figure 1A, formalin-fixed gross specimen). On frozen section, fat lobulation was accentuated by thickened fibrous septa (Figure 1B, circled area in 1A, toluidine blue, ×100) with signet ring-appearing cell clusters (Figure 1C, circled area in 1B, ×400). Despite a quite challenging diagnosis solely based on morphology, their lobular configuration unfavored the diagnosis of peritoneal metastasis. Their adipocytic nature was subsequently confirmed by positive S100 immunostaining and pancytokeratin negative staining (Figure 1D, hematoxylin-eosin-saffron, ×400 and insets). Cytoplasmic lipofuscin pigments are an interesting sign, well preserved by freezing. Body mass index was 14.7 kg/m^2^.

**Figure 1:**
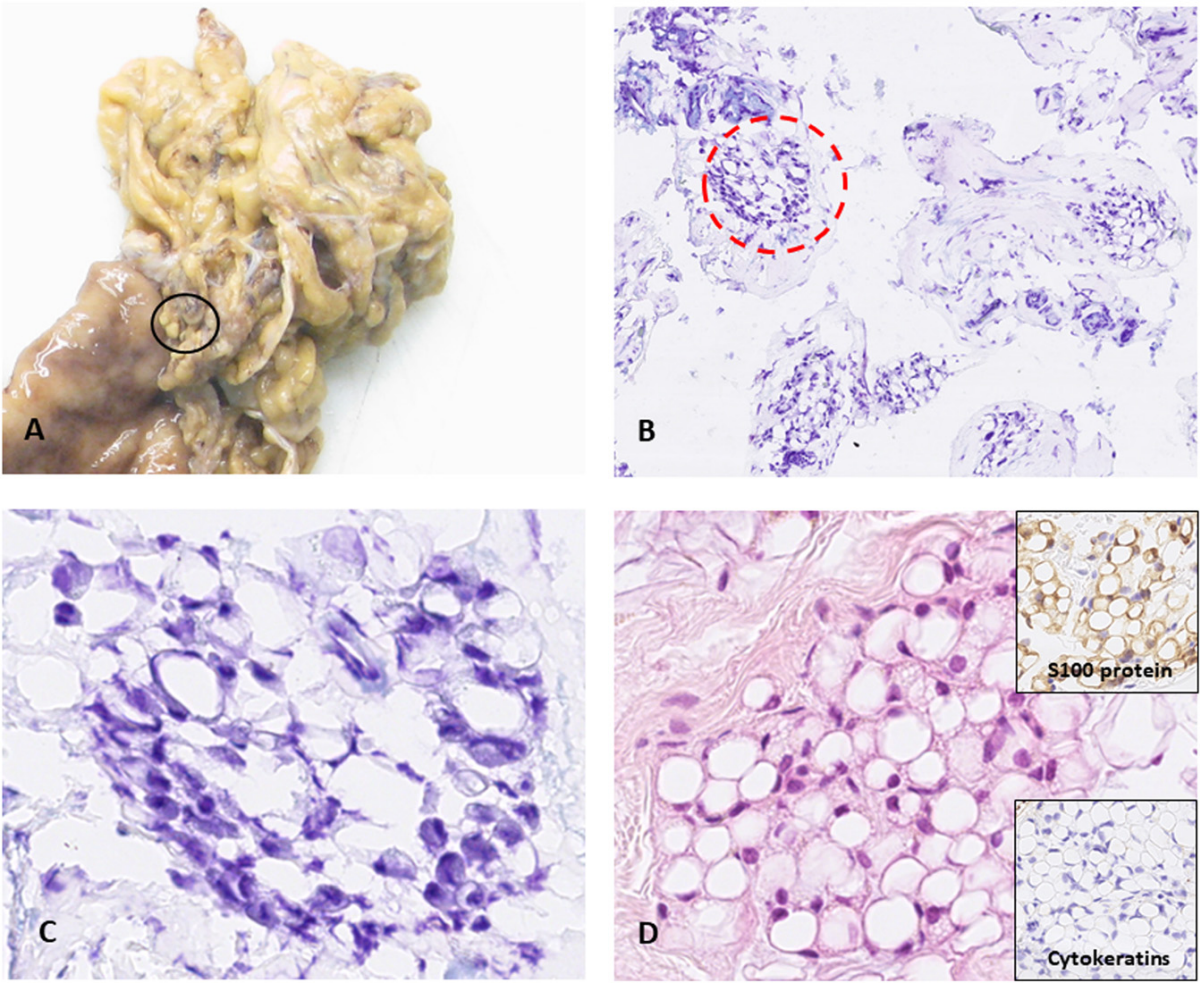
Intraoperative consultation of suspect peritoneal micronodules. (A) Gross specimen of the gastrectomy after formalin fixation: peritoneum of the lesser omentum is involved by micronodules (circled), biopsied for intraoperative consultation. (B) Frozen section showing clusters of small cells circled by fibrous tissue (toluidine blue, ×100). (C) Higher magnification evidencing signet ring-like cells (area circled in B, ×400). (D) Formalin-fixed and paraffin-embedded tissue for definitive diagnosis: signet ring-like cells were proved to be depleted adipocytes in a context of cachexia (hematoxylin-eosin-saffron, ×400), highlighted by pS100 immunostaining, an adipocyte marker (inset).

Cachexia is underestimated in cancer patients [1]. Peritoneal metastases of diffuse-type adenocarcinoma may be overlooked [2], [3]. We emphasize here fat atrophy as a potential false-positive mimic, especially during critical time of surgery, both at the macroscopic and microscopic level.
